# Clinical efficacy of acupuncture for women with PCOS undergoing IVF/ICSI: a meta-analysis of randomized controlled trials

**DOI:** 10.3389/fendo.2026.1845255

**Published:** 2026-05-29

**Authors:** Shanshan Guo, Hong Xia, Weihong Yang, Guangyao Lin, Qianjue Tang, Lianwei Xu

**Affiliations:** 1 Department of Gynecology, Longhua Hospital, Shanghai University of Traditional Chinese Medicine, Shanghai, China; 2 National Clinical Research Center for Obstetrics and Gynecology (Peking University Third Hospital), Beijing, China

**Keywords:** acupuncture, ICSI (intracytoplasmic sperm injection), *in vitro* fertilization, meta-analysis, polycystic ovary syndrome

## Abstract

**Background:**

Infertility constitutes a critical global reproductive health issue, with its overall incidence exhibiting a substantial upward trend. Polycystic ovary syndrome (PCOS) is recognized as a predominant etiological factor in female infertility. Although accumulating clinical evidence suggests that acupuncture may exert therapeutic benefits in improving reproductive outcomes, existing findings remain inconsistent. Accordingly, this meta-analysis was conducted to systematically evaluate current randomized controlled trials (RCTs) and determine whether acupuncture improves clinical outcomes in infertile women with PCOS undergoing *in vitro* fertilization (IVF) or intracytoplasmic sperm injection (ICSI).

**Methods:**

Relevant RCTs investigating the effects of acupuncture in infertile women with PCOS undergoing IVF/ICSI were identified through an exhaustive search of seven databases from inception to March 7, 2026. Forest plots were used to present the pooled evidence. The outcomes comprised one primary outcome and nine secondary outcomes. Risk of bias was appraised using the RoB 2.0 tool. Sensitivity analyses were performed to examine the stability of pooled estimates, while publication bias was assessed with Egger’s and Begg’s tests. Subgroup analyses were also conducted to evaluate differences in therapeutic effects on the primary outcome across acupuncture modalities, number of acupoints, and ovarian stimulation protocols. The certainty of the evidence was assessed utilizing GRADEpro GDT.

**Results:**

Of the 551 initially identified articles, 22 RCTs encompassing 2,299 infertile women with PCOS undergoing IVF/ICSI were included. Meta-analysis demonstrated that acupuncture therapy was associated with a 13% increase in clinical pregnancy rate (CPR) (RD = 0.13, 95% CI: 0.09 to 0.17; *p* < 0.00001) and a 15% increase in live birth rate (RD = 0.15, 95% CI: 0.09 to 0.21; *p* < 0.00001), along with an increased number of optimal embryos (MD = 0.42, 95% CI: 0.17 to 0.66; *p* = 0.0009) and higher E_2_ on the day of hCG (SMD = 0.30, 95% CI: 0.07 to 0.53; *p* = 0.010). Additionally, a reduced total gonadotropin (Gn) dose (MD = -633.45, 95% CI: -1034.65 to -232.24; *p* = 0.002) and shorter duration of Gn use (MD = -0.74, 95% CI: -1.14 to -0.34; *p* = 0.0003) were observed in the acupuncture group. Acupuncture therapy was not associated with an increased incidence of ovarian hyperstimulation syndrome (RD = -0.03, 95% CI: -0.07 to 0.01; *p* = 0.11). Subgroup analysis further suggested that manual acupuncture was associated with a higher clinical pregnancy rate (25%) compared with electroacupuncture (10%). CPR was also higher in the GnRH antagonist protocol (21%) than in the GnRH agonist long protocol (11%) when combined with acupuncture. Sensitivity analyses indicated that the pooled evidence was robust. The overall quality of evidence for all outcomes ranged from very low to moderate.

**Conclusions:**

Evidence from 2,299 infertile women with PCOS suggests that acupuncture interventions may be associated with improved IVF/ICSI outcomes, although findings should be interpreted with caution due to the very low to moderate certainty of evidence. Further rigorous, multicenter studies with more standardized designs and training protocols are therefore warranted to confirm the efficacy of acupuncture in this population.

**Systematic Review Registration:**

https://www.crd.york.ac.uk, identifier CRD420261352381.

## Introduction

The growing prevalence of infertility worldwide has emerged as a global reproductive health issue (1). In 2021, the global prevalence of infertility among females was approximately 3.7%, corresponding to 3,713 cases per 100,000 population, with an estimated 110.1 million women affected worldwide; between 1990 and 2021, the prevalence of female infertility increased by an average of 0.68% per year (2). Regionally, the highest prevalence of infertility was recorded in Côte d’Ivoire (29.6%), followed by China (25.0%), with the trend expected to continue increasing through 2040 (2, 3). Infertility related medical expenses also remain substantial, imposing a considerable economic burden. For every 10,000 affected women, the total financial cost reaches 70.0 million; hospitalizations associated with assisted reproductive technologies (ART) account for the largest proportion of these expenditures, representing 44% of the overall costs (4). Additionally, infertile women are susceptible to a variety of psychological comorbidities, with reported prevalence rates of 57.5% for mild depression, 42.5% for moderate anxiety, and 37.5% for severe stress (5). It is therefore imperative for policymakers to recognize the severity of infertility and prioritize targeted interventions to improve reproductive health. For example, in the context of China’s declining birth rates, the Chinese government has explored insurance reimbursement policies to cover ART services; however, standardized evidence supporting scientific reimbursement thresholds remains insufficient to improve affordability and accessibility (6).

Although infertility has multiple causes, ovulatory disorders account for estimated 25% of all infertile cases; among women with anovulation, 70% are affected by polycystic ovary syndrome (PCOS) (1). The global prevalence of infertility attributable to PCOS rose from 6.00 million people in 1990 to 12.13 million in 2019, with a sharp increase observed across most regions worldwide (7). Fortunately, ART offers a viable pathway to pregnancy for infertile women with PCOS. However, women with PCOS face a dramatically elevated incidence of ovarian hyperstimulation syndrome (OHSS) during ovarian stimulation compared to their counterparts without PCOS (8). Notably, while emerging ovarian stimulation protocols have been developed to improve ART efficacy in women with PCOS, optimizing reproductive outcomes remains a major challenge for clinicians and patients alike. For example, the novel PPOS protocol demonstrates considerable clinical value in elevating clinical pregnancy rate (CPR), cumulative pregnancy rate, and optimal embryos rate, while reducing cycle cancellation rate among women with diminished ovarian reserve (9). Nevertheless, a recent meta-analysis including 2,289 women with PCOS showed that the PPOS protocol may increase CPR; however, it does not significantly elevate the live birth rate, the number of retrieved oocytes, or the number of optimal embryos. In addition, the gonadotropin (Gn) dosage shows a significantly rising trend with the PPOS protocol (10). Recently, numerous clinical trials have reported the value of co-treatments during *in vitro* fertilization (IVF) or intracytoplasmic sperm injection (ICSI). For instance, metformin as a co-treatment with a long GnRH-agonist protocol may raise live birth rates, whereas co-treatment with a short GnRH-antagonist protocol reduces live birth rates (11). Given the limitations of current regimens, complementary approaches such as acupuncture are increasingly applied in the management of women with PCOS undergoing IVF/ICSI.

Acupuncture has been incorporated into the management of reproductive disorders with supporting evidence and is recommended for the management of PCOS based on expert consensus (12–15). Despite the widespread use of acupuncture to improve IVF/ICSI outcomes, the evidence supporting its efficacy in enhancing multiple relevant outcomes remains inconsistent. For example, a randomized controlled trial (RCT) published in 2026 indicated that the findings do not support a superior effect of acupuncture over control in increasing the high-quality embryo rate (16), which is inconsistent with another trial published in 2025 (17). Li et al. recruited 586 women with PCOS and showed that acupuncture significantly increased live birth rate, the number of retrieved oocytes, CPR, and estradiol (E_2_) levels on the day of hCG (18). In contrast, Guo et al. enrolled 100 participants and reported no significant improvements in these reproductive outcomes (19). These inconsistencies in the findings of previous RCTs may be attributed to single-center designs and limited sample sizes, which could compromise the robustness of their conclusions. Therefore, a comprehensive systematic review is warranted. The aim of this review was to systematically analyze existing RCTs and conduct a meta-analysis to quantitatively assess the efficacy and safety of acupuncture in women with PCOS undergoing IVF/ICSI.

## Methods

The PRISMA (Preferred Reporting Items for Systematic Reviews and Meta-Analyses) Statement was followed (20). This meta-analysis has been registered on PROSPERO (No: CRD420261352381).

### Literature search strategy

Seven databases were searched from their inception to March 7, 2026, to identify potentially relevant articles, with the restriction to Chinese or English language. The databases included Wanfang, VIP Information, CNKI, PubMed, the Cochrane Library, Web of Science, and SinoMed. The key search terms were (infertility, polycystic ovary syndrome, PCOS) and (acupuncture, electroacupuncture, assisted reproductive technology, *in vitro* fertilization, intracytoplasmic sperm injection) and (randomized controlled trial). Two reviewers (SSG and HX) independently searched the databases and screened all retrieved abstracts for eligibility (**Supplementary Material 1**). All studies evaluating acupuncture in women with PCOS undergoing IVF/ICSI were retained for full-text review by the same authors. Any disagreements were resolved through prompt consensus with the corresponding author.

### Inclusion and exclusion criteria

The inclusion criteria were as follows: (I) participants were diagnosed with PCOS in accordance with international diagnostic criteria (21, 22); (II) enrolled participants with PCOS underwent IVF or ICSI; (III) the study design was an RCT investigating the effects of acupuncture on reproductive outcomes; (IV) participants were allocated to either a trial group and a control group; (V) the intervention comprised acupuncture, regardless of needling techniques, while control conditions included conventional hormone medication, sham acupuncture, needling at non-acupoints, usual care, or no treatment. Trials were also eligible if acupuncture was administered as an adjuvant intervention before or during ovarian stimulation, provided that both the acupuncture and control groups received identical concomitant background treatment.

The exclusion criteria were as follows: (I) non-needle acupoint interventions similar to acupuncture, such as transcutaneous electrical acupoint stimulation, electrostimulation without needles, and acupressure; (II) not peer-reviewed publications, including dissertations and conference abstracts; (III) duplicate publications or overlapping data, reviews, study protocols, case reports, animal experiments, and meta-analyses; (IV) studies comparing different forms of acupuncture alone, or trials in which the intervention group received additional moxibustion or herbal medicine that was not applied in the control group.

### Data extraction and risk of bias

Data were independently extracted from the included studies by two reviewers (SSG and HX) and double entered into an Excel spreadsheet for analysis. Any disagreements were resolved through consensus. All relevant information was recorded using a standardized data extraction form, covering publication details (first author, publication year, country), participant characteristics and trial design (sample size, age, duration of infertility, BMI, regimen of treatment, ovulation induction protocol, duration of treatment, blinding), as well as the acupuncture protocol (number of acupoints, treatment sessions), outcome measurements, treatment compliance, and adverse events. The primary outcome was CPR. The secondary outcomes included live birth rate, number of oocytes retrieved, cycle cancellation rate, early miscarriage rate, number of optimal embryos, E_2_ on the day of hCG, the rate of OHSS, total dose of Gn used, and duration of Gn used. In addition, each RCT was assessed for risk of bias using the RoB 2.0 tool across six domains. Each domain was rated as having a low risk, high risk, or some concerns (23).

### Statistical analysis

The meta-analysis was performed using Review Manager 5.3 and Stata 15.1. Continuous data were expressed as mean difference (MD) with 95% confidence intervals (CIs). Standardized mean difference (SMD) was applied for continuous data reported in different units. Statistical heterogeneity across trials was evaluated using the I^2^ statistic. An I^2^ value ≤ 50% indicated low to moderate heterogeneity, while an I^2^ value > 50% represented substantial heterogeneity. A random-effects model was adopted for pooling data when the I^2^ value exceeded 50%; if not, a fixed-effect model was used (24). A p value ≤ 0.05 was considered statistically significant. Furthermore, sensitivity analysis was carried out to evaluate the robustness of the overall results by sequentially excluding individual RCTs (25). Subgroup analyses using the leave-one-out approach were performed to identify potential differences in therapeutic effects according to different acupuncture modalities (manual acupuncture *vs*. electroacupuncture), the number of acupoints (≥ 10 *vs*. < 10), and ovarian stimulation protocols (GnRH antagonist protocol *vs.* GnRH agonist long protocol); meanwhile, potential sources of heterogeneity were also explored through subgroup analyses. If heterogeneity remained unexplained after these analyses, it was explicitly acknowledged as a limitation. Publication bias was scrutinized using Begg’s and Egger’s tests when the number of included studies was no fewer than 10. A p value ≥ 0.05 indicated no significant publication bias.

### Overall quality of the evidence

The overall quality of clinical evidence for each pooled outcome was assessed using the GRADE criteria (26), which evaluated study limitations, imprecision, inconsistency, indirectness, and publication bias. Based on these domains, the evidence quality was rated as high, moderate, low, or very low. A “Summary of findings” table was generated using the GRADEpro GDT (https://gdt.gradepro.org).

## Results

### Included articles

**Figure 1:**
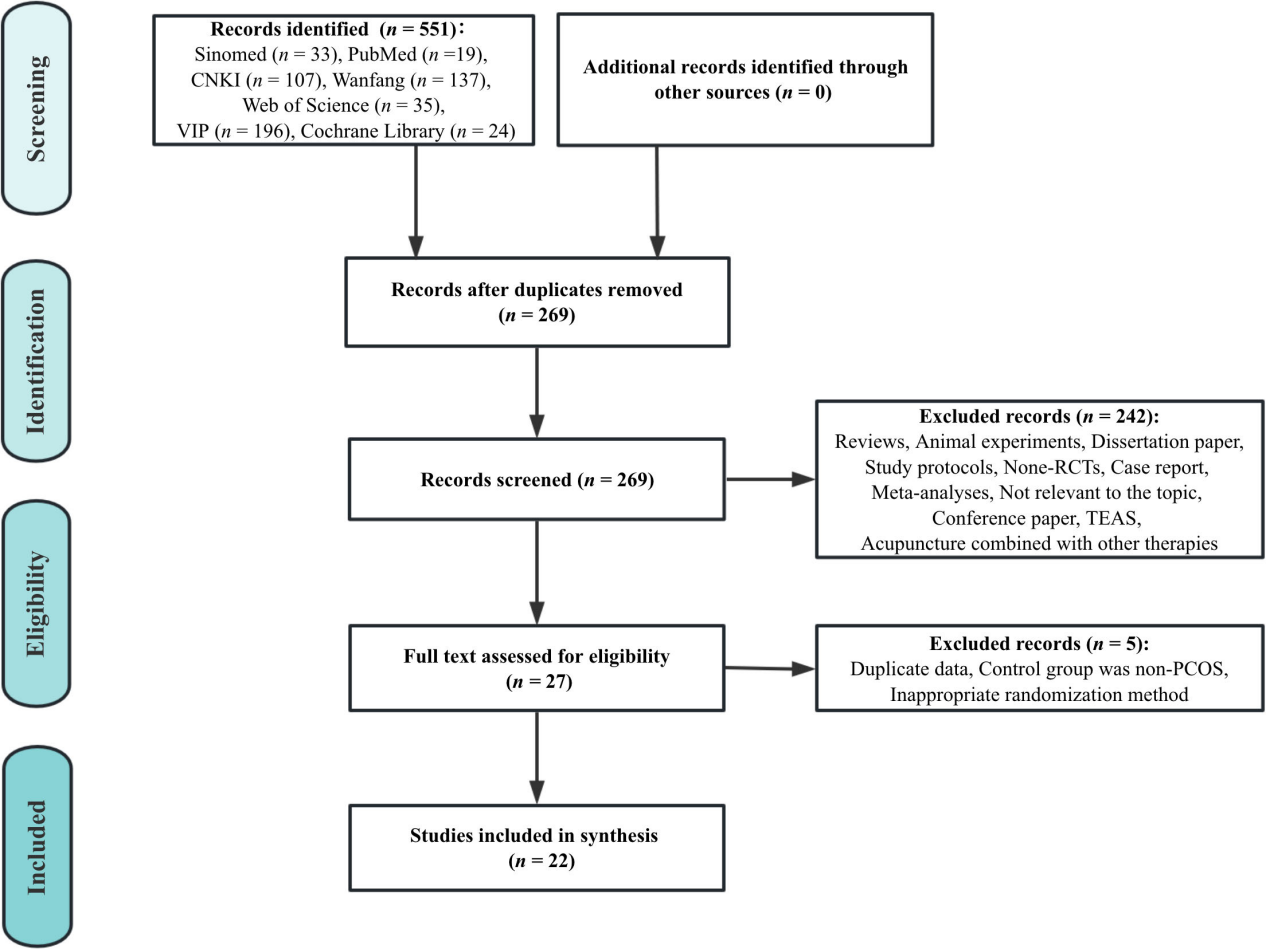
Studies selection flowchart.

The initial literature search yielded 551 potentially relevant titles. Among these, 282 were duplicate records, and 242 were excluded as they met the predefined exclusion criteria. A total of 27 clinical trials underwent full-text evaluation. Of these, 5 studies were excluded owing to overlapping data, non-PCOS study populations in the control group, or inappropriate randomization methods. Ultimately, the remaining 22 RCTs were included in the qualitative synthesis (**Figure 1**).

### Study characteristics

The main baseline characteristics of the included trials are recorded in **Table 1**. This meta-analysis comprised 22 RCTs involving a total of 2,299 women; the sample size of individual studies ranged from 43 to 586, and the studies were published between 2009 and 2026. One study was conducted in Iran, while the remaining 21 studies were performed in China. Of the 22 included trials, 14 applied electroacupuncture, seven adopted manual acupuncture, and one used manual acupuncture combined with traditional Chinese medicine as a co-treatment during IVF/ICSI procedures. The number of acupoints selected per study ranged from five to 20. Regarding ovarian stimulation protocols, one study used a GnRH agonist protocol, 12 employed a GnRH agonist long protocol, seven adopted a GnRH antagonist protocol, one applied a mild stimulation protocol, and one did not report the relevant protocol (**Supplementary Material 2**).

**Table 1:** Characteristics of the included articles.

Study	Year	Sample size (*n*)		Age (years)		Duration of infertility (years)		Outcomes
		Control group	Trial group	Control group	Trial group	Control group	Trial group	
Chen (27)	2009	46	50	32.59 ± 3.92	31.07 ± 4.46	7.08 ± 4.66	6.26 ± 4.31	①③⑦⑧⑩
Li (28)	2009	20	23	32.48 ± 4.45	32.63 ± 4.26	6.75 ± 3.01	6.86 ± 2.62	①③④⑦⑧⑩
Cui (29)	2011	32	34	29.28 ± 3.45	29.29 ± 3.66	4.25 ± 3.01	4.00 ± 2.62	①②③④⑤⑥⑧⑩
Rashidi (30)	2013	31	31	32.10 ± 4.68	31.03 ± 4.82	9.41 ± 4.93	9.09 ± 4.65	①③⑧⑩
Yang (31)	2015	98	102	30.99 ± 4.99	31.24 ± 4.72	4.69 ± 3.41	4.36 ± 3.16	①③④⑤⑥⑧
Li (32)	2015	98	102	31 ± 5	31 ± 5	5 ± 3	5 ± 3	①③④⑤⑦⑧⑨⑩
Liu (33)	2019	30	30	30.00 ± 3.89	28.13 ± 4.32	3.43 ± 2.37	4.67 ± 2.71	③⑥⑦
Cai (34)	2020	42	40	33.39 ± 3.34	33.57 ± 3.20	4.08 ± 3.02	3.90 ± 2.24	①③⑦
Wu (35)	2021	50	50	31 ± 3	31 ± 3	3.71 ± 2.20	3.62 ± 1.97	①③⑦⑨⑩
Xiang (36)	2021	38	38	29.2 ± 5.4	28.4 ± 5.1	3.0 ± 2.2	2.9 ± 1.2	①②③⑥⑨
Guo (19)	2022	50	50	30.88 ± 3.299	30.32 ± 3.292	3.46 ± 2.022	3.08 ± 1.589	①③⑦⑧⑩
Xing (37)	2022	36	36	34.24 ± 5.67	34.85 ± 5.60	4.94 ± 1.76	4.64 ± 1.45	①②⑨
Wu (38)	2022	43	40	29.51 ± 2.96	29.18 ± 5.39	3.19 ± 2.14	3.53 ± 1.75	①②⑦⑩
Liu (39)	2022	30	30	31.67 ± 5.39	32.82 ± 5.43	3.08 ± 2.27	3.11 ± 2.46	①⑥
Ren (40)	2024	30	30	31.87 ± 2.18	32.23 ± 2.78	3.93 ± 0.74	4.00 ± 0.64	①②③⑧⑨
Guan (41)	2024	48	49	31.03 ± 3.56	30.41 ± 2.77	3.35 ± 1.31	3.47 ± 1.16	①②③⑥⑤⑩
Xiao (17)	2025	30	30	31 ± 4	30 ± 3	3.30 ± 2.40	3.23 ± 2.40	③⑦
Pang (42)	2025	45	45	30.97 ± 3.91	30.62 ± 3.88	3.64 ± 2.06	4.51 ± 2.50	②⑥
Yang (43)	2025	32	31	28.90 ± 4.01	29.56 ± 3.25	2.37 ± 0.97	2.63 ± 0.91	⑦
Li (18)	2025	293	293	28.86 ± 3.81	28.86 ± 3.80	4.00 ± 2.40	4.13 ± 2.64	①②③⑦⑥⑤⑨⑩
Xin (44)	2025	33	32	31.74 (29.73, 32.68)	30.84 (30.16, 32.26)	4.00 (2.83, 4.65)	3.05 (2.50, 4.35)	③⑥⑦⑩
Liu (16)	2026	28	28	29.36 ± 3.347	29.86 ± 4.025	2.00 (2)	4.00 (4)	⑥

① Clinical pregnancy rate; ② Live birth rate; ③ Number of oocytes retrieved; ④ Cycle cancellation rate; ⑤ Early miscarriage rate; ⑥ Number of optimal embryos; ⑦ E_2_ on the day of hCG; ⑧ The rate of ovarian hyperstimulation syndrome; ⑨ Total dose of gonadotropin used; ⑩ Duration of gonadotropin used.

### Risk of bias

**Figure 2:**
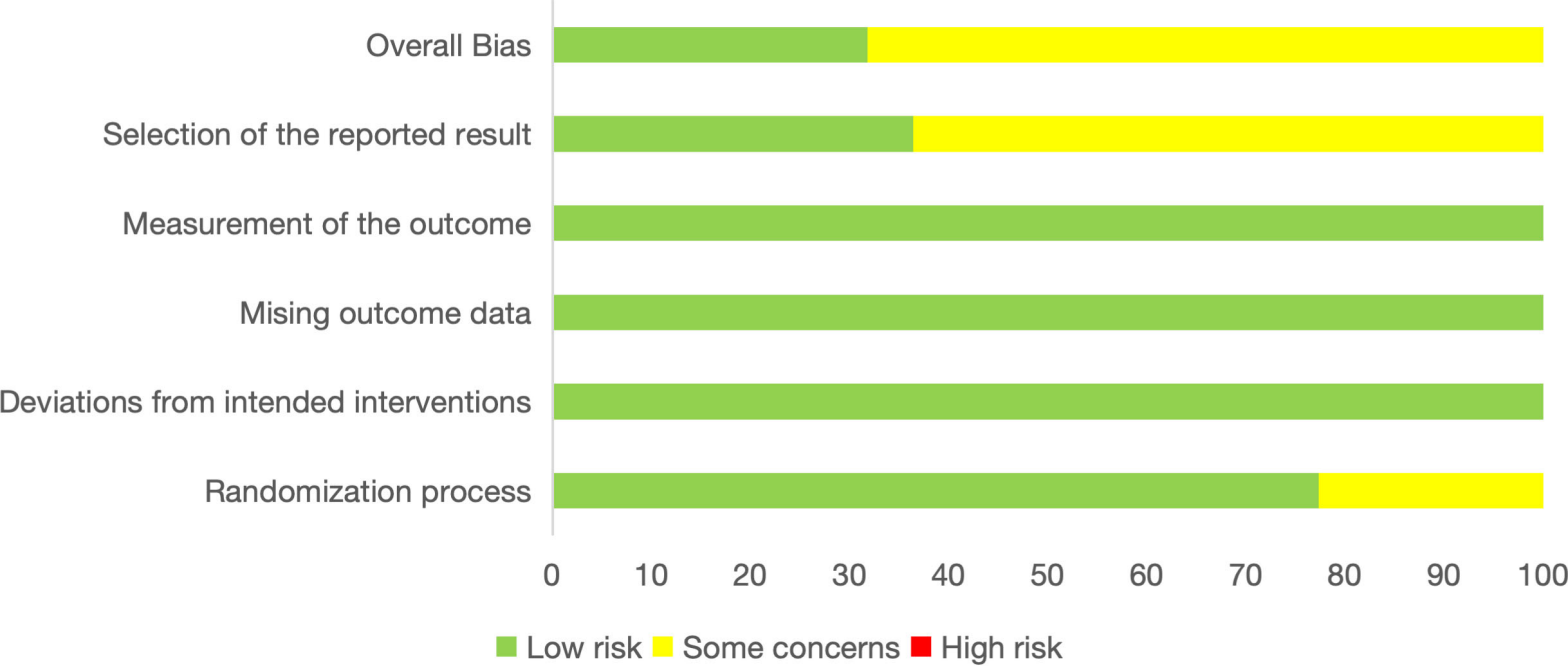
Risk of bias assessment.

All included records were identified as RCTs. Apart from four trials (27, 28, 30, 38), the remaining studies described their randomization procedures in detail and were rated as having low risk. Two trials (16, 44) used participant blinding and four trials (16, 38, 42, 44) implemented blinded outcome assessment. Most studies were judged as low risk of bias, although some methodological concerns were noted. Furthermore, seven RCTs (16, 17, 30, 36, 39, 42, 43) showed no selective reporting, as their trial protocols had been formally registered. Collectively, the main sources of bias were present in the domains of randomization process and selection of the reported results. (**Figure 2**).

### Primary outcome measurements

**Figure 3:**
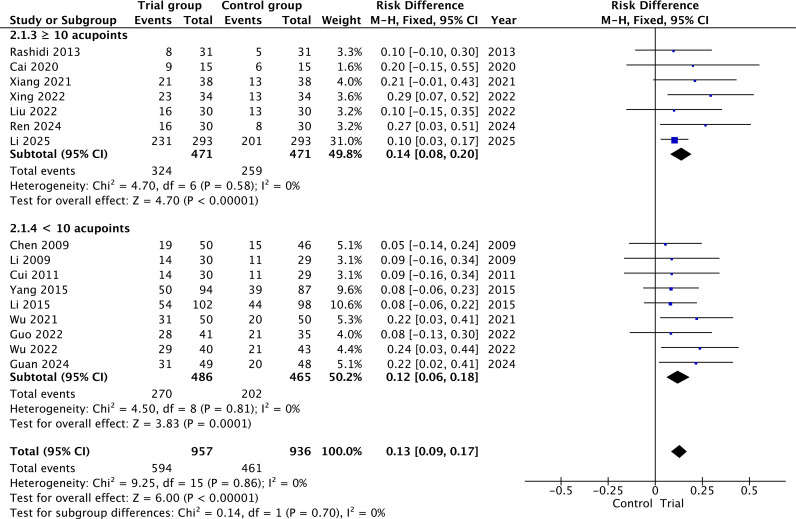
Forest plot of CPR.

16 studies involving 1,893 participants compared the effect of acupuncture as a co-intervention on CPR among women with PCOS undergoing IVF/ICSI. The pooled analysis showed that acupuncture was associated with a notable improvement in CPR (RD = 0.13, 95% CI: 0.09 to 0.17, I^2^ = 0%, *p* < 0.00001) compared to the control group (**Figure 3**) (**Table 2**). The pooled results remained robust following sensitivity analysis.

**Table 2:** Summary of acupuncture treatment for IVF/ICSI outcomes.

Clinical outcomes	Cases (*n*)	RD/MD/SMD 95% CI	*P*	I^2^ (%)	Model
Primary outcomes					
CPR	1,893	0.13 [0.09, 0.17]	< 0.00001	0	Fixed
Secondary outcomes					
Live birth rate	1,027	0.15 [0.09, 0.21]	< 0.00001	0	Fixed
Number of oocytes retrieved	1,888	0.71 [-0.30, 1.71]	0.17	80	Random
Cycle cancellation rate	519	-0.02 [-0.07, 0.03]	0.52	0	Fixed
Early miscarriage rate	834	-0.03 [-0.06, 0.01]	0.13	0	Fixed
Number of optimal embryos	833	0.42 [0.17, 0.66]	0.0009	81	Random
E_2_ on the day of hCG	1,538	0.30 [0.07, 0.53]	0.010	76	Random
The rate of OHSS	897	-0.03 [-0.07, 0.01]	0.11	0	Fixed
Total dose of Gn used	580	-633.45 [-1034.65, -232.24]	0.002	94	Random
Duration of Gn used	1,433	-0.74 [-1.14, -0.34]	0.0003	65	Random
Subgroup analysis					
CPR (Manual acupuncture)	341	0.25 [0.14, 0.35]	< 0.00001	0	Fixed
CPR (Electroacupuncture)	1,552	0.10 [0.06, 0.15]	< 0.0001	0	Fixed
CPR (≥ 10 acupoints)	942	0.14 [0.08, 0.20]	< 0.00001	0	Fixed
CPR (< 10 acupoints)	951	0.12 [0.06, 0.18]	0.0001	0	Fixed
CPR (Acupuncture + GnRH antagonist protocol)	203	0.21 [0.08, 0.35]	0.002	0	Fixed
CPR (Acupuncture + GnRH agonist long protocol)	1,622	0.11 [0.07, 0.16]	< 0.00001	0	Fixed

### Secondary outcome measurements

**Figure 4:**
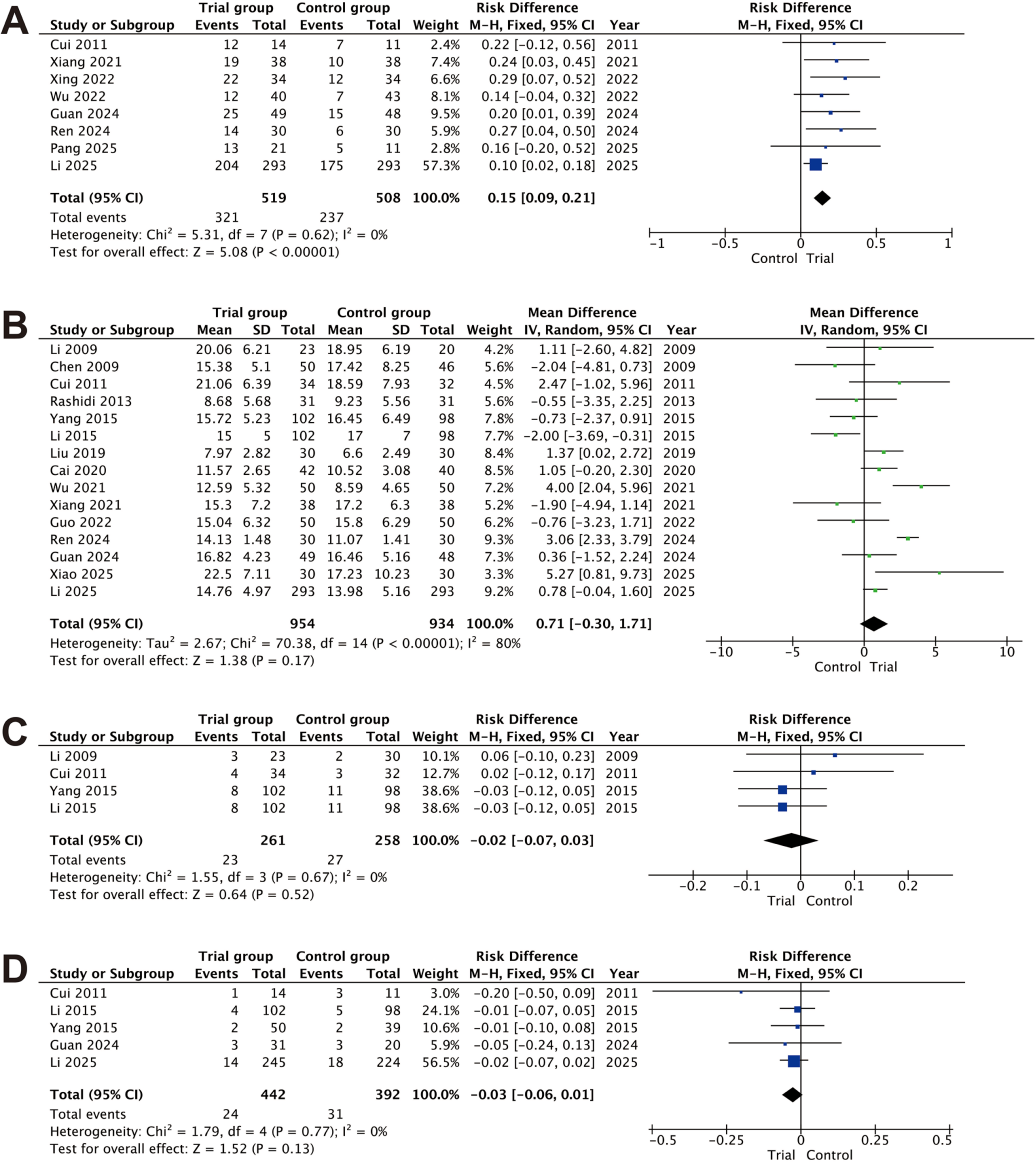
Forest plot of live birth rate **(A)**, number of oocytes retrieved **(B)**, cycle cancellation rate **(C)**, early miscarriage rate **(D)**.

Eight studies involving 1,027 participants evaluated the impact of acupuncture on the live birth rate. Acupuncture as an adjuvant treatment during IVF/ICSI was associated with a considerable increase in live birth rate (RD = 0.15, 95% CI: 0.09 to 0.21, I^2^ = 0%, *p* < 0.00001) (**Figure 4A**). Nevertheless, no statistical differences were observed in the number of oocytes retrieved (MD = 0.71, 95% CI: -0.30 to 1.71, I^2^ = 80%, *p* = 0.17), cycle cancellation rate (RD = -0.02, 95% CI: -0.07 to 0.03, I^2^ = 0%, *p* = 0.52), and early miscarriage rate (RD = -0.03, 95% CI: -0.06 to 0.01, I^2^ = 0%, *p* = 0.13) following acupuncture intervention (**Figures 4B–D**). Sensitivity analysis confirmed that no single study influenced the pooled estimates.

**Figure 5:**
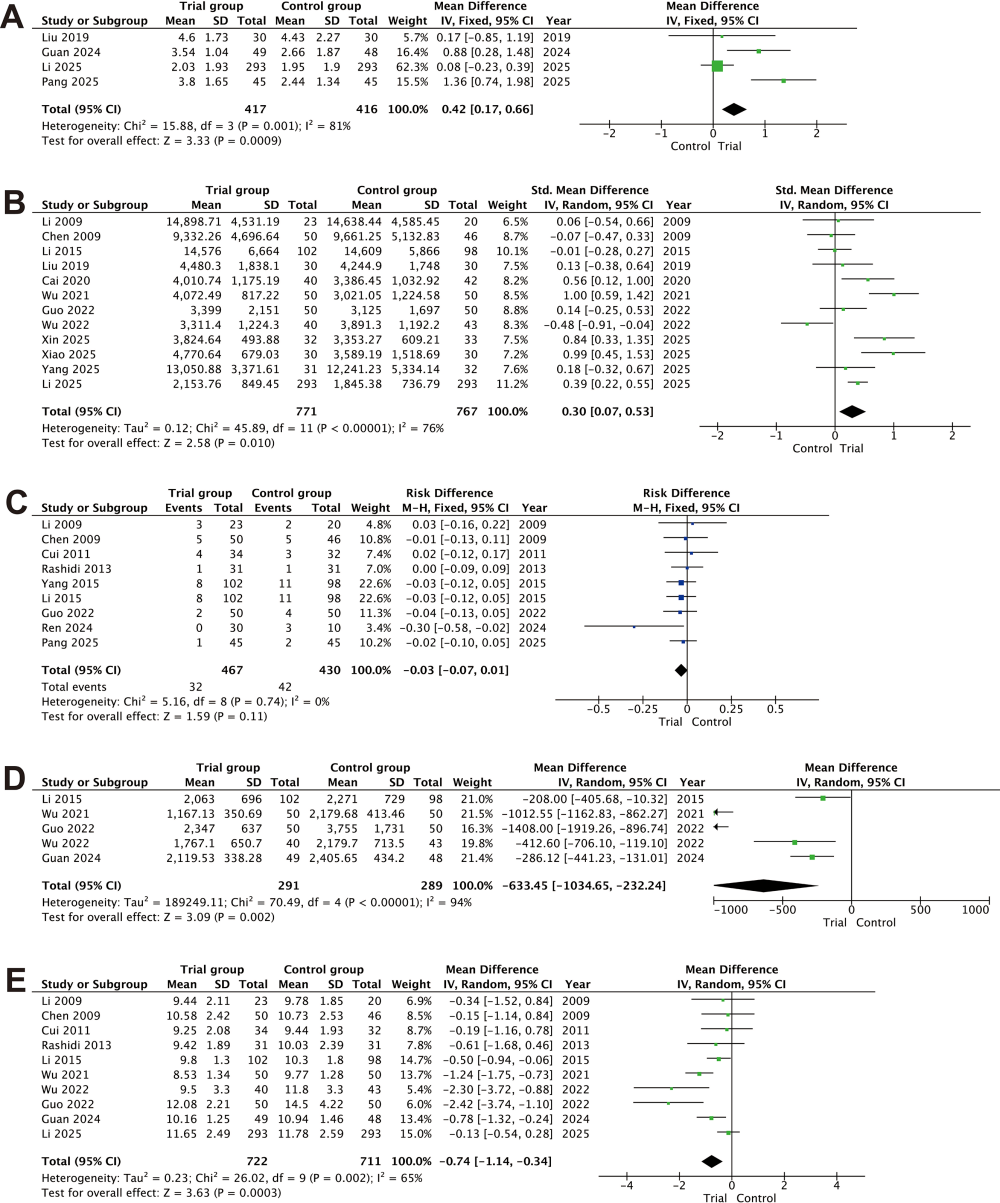
Forest plot of the number of optimal embryos **(A)**, E_2_ on the day of HCG **(B)**, the rate of OHSS **(C)**, total dose of Gn used **(D)**, duration of Gn used **(E)**.

With regard to the number of optimal embryos, data from 833 participants indicated that acupuncture was associated with a greater number of optimal embryos (MD = 0.42, 95% CI: 0.17 to 0.66, I^2^ = 81%, *p* = 0.0009) (**Figure 5A**). For E_2_ on the day of hCG, pooled analyses of 12 RCTs revealed a significant correlation between acupuncture and this outcome (SMD = 0.30, 95% CI: 0.07 to 0.53, I^2^ = 76%, *p* = 0.010) (**Figure 5B**). Notably, although E_2_ levels on the day of hCG were elevated, the rate of OHSS showed no significant increase (RD = -0.03, 95% CI: -0.07 to 0.01, I^2^ = 0%, *p* = 0.11) (**Figure 5C**). Furthermore, the current data provided evidence that acupuncture was associated with reductions in both the total dose of Gn used (MD = -633.45, 95% CI: -1034.65 to -232.24, I^2^ = 94%, *p* = 0.002) (**Figure 5D**) and the duration of Gn used (MD = -0.74, 95% CI: -1.14 to -0.34, I^2^ = 65%, *p* = 0.0003) (**Figure 5D**) during ovarian stimulation. The results remained unchanged after sensitivity analysis. **Table 2** presents the results mentioned above.

### Adverse event reporting

Four studies (37, 38, 40, 42) reported adverse events related to acupuncture treatment. Among them, three trials (37, 38, 42) documented that no adverse events occurred during the trial periods, while one study (40) noted mild adverse events in the acupuncture group, including breast tenderness, dizziness, and nausea.

### Subgroup analysis

Subgroup analyses were conducted to ascertain whether the effects of acupuncture on the primary outcome, CPR, were associated with different acupuncture modalities (manual acupuncture *vs*. electroacupuncture), the number of acupoints (≥ 10 *vs*. < 10), and ovarian stimulation protocols (GnRH antagonist protocol *vs.* GnRH agonist long protocol).

**Figure 6:**
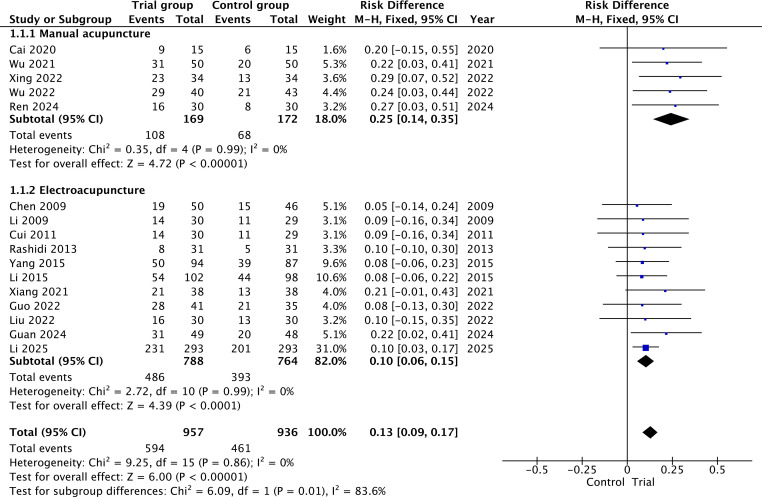
Subgroup analysis of acupuncture modalities (manual acupuncture *vs*. electroacupuncture) for CPR.

Pooled results indicated that manual acupuncture (RD = 0.25, 95% CI: 0.14 to 0.35, I^2^ = 0%, *p* < 0.00001) was associated with a higher CPR compared with electroacupuncture (RD = 0.10, 95% CI: 0.06 to 0.15, I^2^ = 0%, *p* < 0.0001) (**Figure 6**) (**Table 2**).

**Figure 7:**
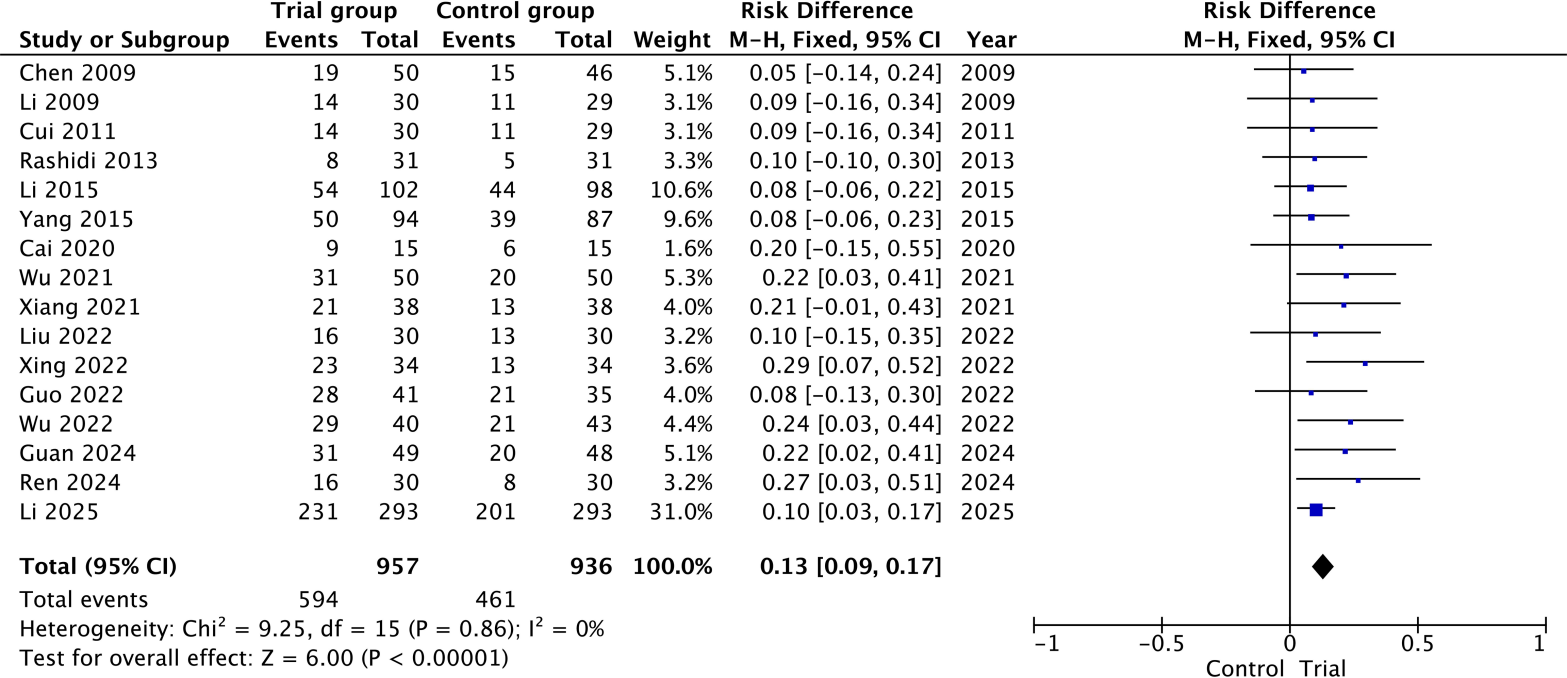
Subgroup analysis of the number of acupoints (≥ 10 *vs*. < 10) for CPR.

The number of acupuncture acupoints varied across the included trials; thus, this meta-analysis adopted 10 as the cutoff value to explore potential therapeutic differences between regimens using ≥ 10 acupoints and those using fewer than 10 acupoints. The available evidence indicated no significant difference in CPR between women with PCOS undergoing IVF/ICSI receiving acupuncture with ≥ 10 acupoints (RD = 0.14, 95% CI: 0.08 to 0.20, I^2^ = 0%, *p* < 0.00001) and those treated with fewer than 10 acupoints (RD = 0.12, 95% CI: 0.06 to 0.18, I^2^ = 0%, *p* = 0.0001) (**Figure 7**) (**Table 2**).

**Figure 8:**
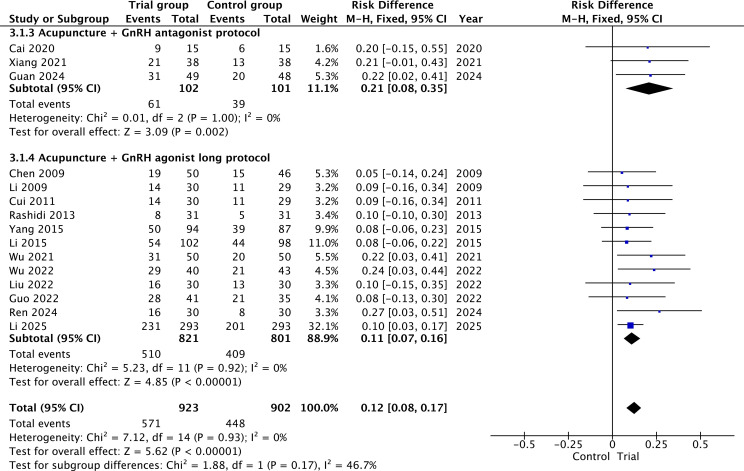
Subgroup analysis of the ovarian stimulation protocols (GnRH antagonist protocol *vs.* GnRH agonist long protocol) for CPR.

Furthermore, one study (37) was excluded from the analysis of the effects of acupuncture combined with different ovarian stimulation protocols, as it failed to report the detailed protocol. Adjuvant acupuncture combined with the GnRH antagonist protocol (RD = 0.21, 95% CI: 0.08 to 0.35, I^2^ = 0%, *p* = 0.002) was associated with a higher CPR compared with the GnRH agonist long protocol (RD = 0.11, 95% CI: 0.07 to 0.16, I^2^ = 0%, *p* < 0.00001) (**Figure 8**) (**Table 2**).

To investigate the sources of heterogeneity, we also performed subgroup analyses for the following five outcomes with high heterogeneity. For the outcome of total dose of Gn used, all included studies used fewer than 10 acupoints; thus, subgroup analysis based on the number of acupoints was not performed for this outcome. **Table 3** indicates that heterogeneity was not substantially reduced in most subgroup analyses, suggesting that the observed heterogeneity may be attributable to other unmeasured factors, such as differences in infertility duration, participant age, or BMI.

**Table 3:** Summary of subgroup analysis for outcomes with high heterogeneity.

Clinical outcomes	Cases (*n*)	MD/SMD 95% CI	*P*	I^2^ (%)	Model
Number of oocytes retrieved					
Manual acupuncture	302	2.30 [1.04, 3.56]	0.0003	76	Random
Electroacupuncture	1,586	-0.16 [-1.14, 0.82]	0.75	53	Random
≥ 10 acupoints	866	0.89 [-0.60, 2.39]	0.24	85	Random
< 10 acupoints	1,022	0.65 [-0.73, 2.03]	0.35	74	Random
Acupuncture + GnRH antagonist protocol	255	0.34 [-1.02, 1.70]	0.62	37	Random
Acupuncture + GnRH agonist long protocol	1,513	0.57 [-0.82, 1.96]	0.42	85	Random
Number of optimal embryos					
Manual acupuncture	60	0.17 [-0.85, 1.19]	0.74	NA	Random
Electroacupuncture	773	0.74 [-0.08, 1.55]	0.08	87	Random
≥ 10 acupoints	586	0.08 [-0.23, 0.39]	0.61	NA	Fixed
< 10 acupoints	247	0.97 [0.57, 1.37]	< 0.00001	49	Fixed
Acupuncture + GnRH antagonist protocol	187	1.11 [0.68, 1.55]	< 0.00001	15	Fixed
Acupuncture + GnRH agonist long protocol	586	0.08 [-0.23, 0.39]	0.61	NA	Fixed
E_2_ on the day of hCG					
Manual acupuncture	388	0.28 [-0.23, 0.79]	0.28	84	Random
Electroacupuncture	1,149	0.30 [0.05, 0.56]	0.02	71	Random
≥ 10 acupoints	668	0.41 [0.25, 0.56]	< 0.00001	0	Random
< 10 acupoints	870	0.36 [0.10, 0.62]	0.007	72	Random
Acupuncture + GnRH antagonist protocol	210	0.52 [0.16, 0.88]	0.004	41	Random
Acupuncture + GnRH agonist long protocol	1,208	0.16 [-0.14, 0.46]	0.30	81	Random
Total dose of Gn used					
Manual acupuncture	183	-491.79 [-625.55, -358.03]	< 0.00001	0	Random
Electroacupuncture	397	-541.31 [-966.15, -116.46]	0.01	89	Random
Acupuncture + GnRH antagonist protocol	97	-286.12 [-441.23, -131.01]	0.0003	NA	Random
Acupuncture + GnRH agonist long protocol	483	-557.79 [-876.18, -239.39]	0.0006	85	Random
Duration of Gn used					
Manual acupuncture	183	-1.36 [-1.85, -0.88]	< 0.00001	47	Fixed
Electroacupuncture	1,250	-0.46 [-0.69, -0.23]	0.0001	47	Fixed
≥ 10 acupoints	648	-0.19 [-0.58, 0.19]	0.33	0	Random
< 10 acupoints	785	-0.87 [-1.32, -0.42]	0.0002	62	Random
Acupuncture + GnRH antagonist protocol	97	-0.78 [-1.32, -0.24]	0.005	NA	Random
Acupuncture + GnRH agonist long protocol	1,336	-0.76 [-1.22, -0.30]	0.001	69	Random

NA, not applicable.

### Publication bias

Publication bias represents a major threat to evidence synthesis and the validity of meta-analyses. Hence, Begg’s and Egger’s tests were performed to assess the potential risk of publication bias. The results indicated no significant publication bias for the outcomes of E_2_ on the day of hCG and duration of Gn used (*p* > 0.05). However, significant publication bias was observed for CPR and number of oocytes retrieved (*p <* 0.05) (**Table 4**). These findings suggest that the pooled estimates for the latter outcomes should be interpreted with caution.

**Table 4:** Begg’s and Egger’s tests for publication bias.

Outcomes	Begg’s test	Egger’s test
CPR	0.024	0.001
Number of oocytes retrieved	0.029	0.219
E_2_ on the day of hCG	0.837	0.515
Duration of Gn used	0.721	0.085

### Certainty of the evidence

The overall certainty of evidence ranged from very low to moderate. With respect to inconsistency, the comparisons for number of oocytes retrieved, number of optimal embryos, E_2_ level on hCG day, total Gn dose, and duration of Gn use were downgraded by one level owing to significant heterogeneity (I^2^ > 50%). Five comparisons (cycle cancellation rate, early miscarriage rate, number of optimal embryos, OHSS rate, and total dose of Gn used) were also downgraded due to small sample sizes (*n* < 1,000). Furthermore, other major considerations applied to these comparisons, including CPR, live birth rate, number of oocytes retrieved, cycle cancellation rate, early miscarriage rate, number of optimal embryos, OHSS rate, and total Gn dose, as for these outcomes either publication bias existed or Begg’s and Egger’s tests could not be performed due to fewer than ten studies being available (**Table 5**).

**Table 5:** Summary of results for GRADE ratings.

Outcomes	Absolute effects (95% CI)	Relative effects (95% CI)	Number of cases	Quality of the evidence (GRADE)
CPR	13 more per 100 (from 8 more to 18 more)	RR 1.26 (1.17 to 1.37)	1,893	⊕⊕⊕⚪^3^ Moderate
Live birth rate	15 more per 100 (from 8 more to 22 more)	RR 1.32 (1.18 to 1.47)	1,027	⊕⊕⊕⚪^3^ Moderate
Number of oocytes retrieved	MD 0.71 higher (0.3 lower to 1.71 higher)	–	1,888	⊕⊕⚪⚪^1,3^ Low
Cycle cancellation rate	2 fewer per 100 (from 5 fewer to 5 more)	RR 0.84 (0.50 to 1.43)	519	⊕⊕⚪⚪^2,3^ Low
Early miscarriage rate	3 fewer per 100 (from 5 fewer to 1 more)	RR 0.65 (0.37 to 1.13)	834	⊕⊕⚪⚪^2,3^ Low
Number of optimal embryos	MD 0.42 higher (0.17 higher to 0.66 higher)	–	833	⊕⚪⚪⚪^1,2,3^ Very low
E_2_ on the day of hCG	SMD 0.3 SD higher (0.07 higher to 0.53 higher)	–	1,538	⊕⊕⊕⚪^1^ Moderate
The rate of OHSS	3 fewer per 100 (from 5 fewer to 1 more)	RR 0.70 (0.45 to 1.07)	897	⊕⊕⚪⚪^2,3^ Low
Total dose of Gn used	MD 633.45 lower (1034.65 lower to 232.24 lower)	–	580	⊕⚪⚪⚪^1,2,3^ Very low
Duration of Gn used	MD 0.74 lower (1.14 lower to 0.34 lower)	–	1,433	⊕⊕⊕⚪^1^ Moderate

^1^ Downgraded one level for serious inconsistency: I^2^ > 50%;.

^2^ Downgraded one level for serious imprecision: very small participants (*n* < 1,000);

^3^ Downgraded one level for other considerations: presence of publication bias, or inability to estimate publication bias.

## Discussion

Given the suboptimal reproductive outcomes observed in women with PCOS undergoing IVF/ICSI, numerous clinicians have sought to explore feasible and novel strategies for improving clinical pregnancy outcomes. Acupuncture, a non-pharmacological approach used alongside IVF/ICSI, has been shown to exert effects through multiple underlying mechanisms. A previous study (45) indicated that acupuncture serves as a beneficial therapy for improving ovulatory dysfunction in women with PCOS by downregulating LncMEG3, thereby suppressing the PI3K/AKT/mTOR pathway, normalizing granulosa cell proliferation, and reducing granulosa cell autophagy. Also, bile acid metabolism has recently been found to play a pivotal role in the pathogenesis of PCOS. Acupuncture may confer therapeutic benefits in women with PCOS by modulating levels of taurocholic acid and lithocholic acid, as well as regulating the FXR signaling pathway, as verified in both women with PCOS and rat models (46). In addition, a recent study using *Rax-CreER*^T2^ transgenic mouse models showed that acupuncture produces therapeutic effects on PCOS by targeting Itgb1 and the hypothalamic GnRH-tanycyte unit. These findings offer novel insights into the underlying mechanisms of acupuncture for PCOS (47). Notably, oocyte quality, gut dysbiosis, and metabolic profiles have become major research focuses in recent years. PCOS entails dysregulation of reproductive endocrine and metabolic processes (16). An RCT including 60 women with PCOS undergoing IVF demonstrated that acupuncture combined with IVF significantly reduced the abundance of Escherichia-Shigella, regulated glucose and lipid metabolism, and yielded potential benefits in improving embryonic developmental potential and oocyte quality (16). Another study focusing on the targets of androgen response in the brain demonstrated that acupuncture can regulate the kisspeptin-GnRH/LH circuit by modulating androgen activity, such as reducing the number of AR/Kiss1-positive cells, decreasing the protein expression levels of Kiss1, and Ar, along with increasing the count of ERα/Kiss1-positive cells (48).

### Main results

This study reviewed the clinical value of acupuncture in women with PCOS undergoing IVF/ICSI. A total of 22 RCTs comprising 2,299 participants were ultimately included into this study. This meta-analysis showed that acupuncture was associated with a 13% increase in CPR and a 15% increase in live birth rate. Although several previous meta-analyses (49, 50) could not exclude clinically relevant differences regarding live birth and CPR following acupuncture intervention, their conclusions may not be generalizable to women with PCOS undergoing IVF/ICSI. Therefore, the present study may provide a potential combined treatment option for clinical practice. In addition, improving the yield of optimal embryos remains a major clinical challenge for women with PCOS receiving IVF. A recent clinical trial comparing four ovarian stimulation protocols regarding the production of optimal embryos suggested that only one protocol achieved favorable outcomes in this respect (51). Notably, the present meta-analysis provides evidence that acupuncture co-treatment may significantly increase the number of optimal embryos. Furthermore, in ovarian stimulation protocols, achieving higher E_2_ levels on the day of hCG is challenging when reducing the total Gn dose or shortening the duration of Gn treatment. Meanwhile, higher Gn exposure is frequently associated with an elevated risk of OHSS. Of note, this meta-analysis revealed that among women with PCOS receiving IVF/ICSI, adjuvant acupuncture was associated with higher E_2_ on the day of hCG, along with a lower total Gn dose and a shorter duration of Gn administration. A previous study of 368 cases found that acupuncture could reduce the incidence of OHSS, which is inconsistent with our findings based on 897 women. This discrepancy may result from the substantial difference in sample sizes, and the true effect of acupuncture may not have been accurately estimated due to low statistical power. Subgroup analysis further suggested that manual acupuncture was associated with a higher CPR (25%) compared with electroacupuncture (10%). At the same time, CPR was also higher in the GnRH antagonist protocol (21%) than in the GnRH agonist long protocol (11%) when combined with acupuncture. These findings are hypothesis-generating and based on indirect cross-study comparisons. Given the limited certainty of evidence, further high-quality multicenter RCTs are warranted. In particular, it is important to interpret these findings in light of the GRADE certainty ratings. The certainty of evidence was moderate for CPR, live birth rate, E_2_ on the day of hCG, and duration of Gn use; low for number of oocytes retrieved, cycle cancellation rate, early miscarriage rate, and OHSS rate; and very low for number of optimal embryos and total dose of Gn used. Therefore, the most robust evidence supports the beneficial effects of acupuncture on CPR and live birth rate, whereas conclusions regarding optimal embryos and total dose of Gn used remain preliminary.

### Differences with other studies

In 2019, a meta-analysis including 1,546 women with PCOS who did not undergo IVF/ICSI confirmed that acupuncture may improve the number of intermenstrual days, but did not improve multiple pregnancy rate, live birth rate, CPR, ovulation rate, or miscarriage rate (49). In contrast, the present meta-analysis focused on the specific population of women with PCOS undergoing IVF/ICSI.

In addition, a similar meta-analysis published in 2017 evaluated the effects of acupuncture in women with PCOS undergoing IVF/ICSI and illustrated the benefits of acupuncture in improving CPR and ongoing pregnancy rates, as well as reducing the risk of OHSS (52). However, several differences exist between the present meta-analysis and prior study. First, the prior study only searched databases up to October 2015 and included four RCTs involving 430 participants in its analysis. In contrast, this meta-analysis searched for the latest studies across seven databases and included 22 RCTs involving 2,299 participants, which may make the pooled evidence from this meta-analysis more robust. Second, the present meta-analysis qualitatively evaluated ten clinical outcomes, including one primary outcome and nine secondary outcomes, whereas the earlier study reported four outcomes with relatively limited sample cases. This may not systematically reflect the true clinical value of acupuncture in routine practice. For example, in the previous meta-analysis, findings based on 66 participants indicated that acupuncture failed to improve the live birth rate. Conversely, the present meta-analysis, comprising 1,027 participants, showed that acupuncture was associated with a significant improvement in this outcome. Third, we employed Begg’s and Egger’s tests, GRADE assessment, and sensitivity analysis to evaluate the robustness and certainty of the pooled evidence, which were not used in the existing meta-analysis. Fourth, our study further conducted subgroup analyses to explore differences in therapeutic effects on the primary outcome across different acupuncture modalities, number of acupoints, and ovarian stimulation protocols, and found that there may be differences in pregnancy rates associated with different acupuncture modalities and ovarian stimulation protocols. Thus, the results of this meta-analysis provide more comprehensive evidence regarding the clinical value of adjuvant acupuncture in women with PCOS undergoing IVF/ICSI.

### Limitations of this study

Some limitations of this analysis should be acknowledged. First, IVF/ICSI outcomes may correlate with participants’ age and the duration of infertility; accordingly, younger women with a shorter duration of infertility tend to achieve better reproductive outcomes. However, none of the included RCTs explored the differential effects of acupuncture co-treatment. Second, although the present meta-analysis included 2,299 participants, relatively small sample sizes were available for the assessment of certain outcomes. For instance, several trials reported Gn dosage in vial quantities rather than standardized uniform units. Accordingly, only unified measurements involving 580 participants could be applied for further analysis. Third, apart from OHSS, several acupuncture-related adverse events were not reported across the 18 included studies. Hence, a systematic quantitative analysis regarding the safety of acupuncture co-treatment during IVF/ICSI could not be performed in the present analysis. Fourth, although subgroup analyses were performed in this meta-analysis to explore potential sources of heterogeneity (e.g., acupuncture modalities, number of acupoints, ovarian stimulation protocols), these analyses did not substantially reduce the overall heterogeneity. This indicates that the observed heterogeneity could not be fully explained by the prespecified factors. Under such conditions, pooled estimates from random-effects models should be interpreted with particular caution. The presence of high heterogeneity suggests that the true treatment effects may vary considerably across different populations, acupuncture protocols, or clinical settings. Therefore, we emphasize that for outcomes with I^2^ > 50%, the pooled estimates are presented as exploratory rather than confirmatory, and we avoid overemphasis on their numerical magnitude. Future studies with standardized acupuncture protocols and larger sample sizes are needed to obtain more stable and generalizable estimates. Future studies should adopt more standardized acupuncture protocols to minimize clinical heterogeneity. Fifth, publication bias was detected for CPR and number of oocytes retrieved, together with some concerns regarding risk of bias, which may have inflated the pooled effect estimates and downgraded the certainty of these findings. Sixth, many of the included RCTs lacked key safeguards such as allocation concealment and blinding challenges inherent to acupuncture trials. Under RoB 2.0 standards, even objective outcomes may be susceptible to overestimation due to performance or detection bias introduced through differential clinical management or selective outcome reporting. This concern is particularly relevant for outcomes such as CPR and live birth rates, where the moderate GRADE certainty of evidence does not fully mitigate the risk of bias. For outcomes with low or very low GRADE certainty (e.g., number of optimal embryos), the potential for overestimation is even greater. Therefore, although acupuncture may be associated with beneficial effects, the pooled effect sizes should be interpreted with caution.

### Implications for future study

First, during the literature screening process, several excluded studies claimed to adopt a randomized design; however, their randomization procedures were inappropriate. For instance, some trials allocated participants based on odd or even numbers without specifying how these numbers were generated. Therefore, further clinical trials with rigorous methodology and standardized randomization are warranted, preferably designed in accordance with the STRICTA guidelines and the CONSORT statement. Second, all included studies were single-center trials; only four mentioned blinding procedures, and seven registered their study protocols in advance. Therefore, future multicenter trials adopting sham or placebo acupuncture are warranted. Third, although an increasing number of studies have explored the potential mechanisms of acupuncture for treating women with PCOS, evidence regarding its direct effects on ovarian stimulation and ovarian response remains scarce. Further research clarifying the specific underlying mechanisms of acupuncture would be valuable. Fourth, of the 22 included RCTs, 21 were conducted in China and only one in Iran. This geographic concentration substantially limits the generalizability of the findings to other populations with different ethnic backgrounds, cultural biases, dietary habits, and healthcare systems. These factors may influence treatment effects and are not easily replicable across diverse healthcare systems. Thus, the evidence was derived almost exclusively from Chinese populations, and the findings should not be directly generalized to non-Chinese populations without validation in multinational, multicenter studies. Future research should prioritize recruitment of diverse ethnic and geographic populations to assess external validity. Fifth, the 22 RCTs differed considerably in acupuncture modality (manual acupuncture *vs*. electroacupuncture), treatment frequency (ranging from once daily to three times weekly), timing of intervention (before or during ovarian stimulation), and number of acupoints (ranging from 5 to 20). This clinical heterogeneity limits the interpretability of the pooled estimates, as it remains unclear which specific protocol confers the greatest benefit. Furthermore, the control conditions were equally heterogeneous, including sham acupuncture, no treatment, and conventional care alone. Pooling such diverse control conditions may inflate effect sizes, as the true magnitude of the placebo or contextual effect cannot be reliably estimated. Consequently, the present findings should be interpreted as hypothesis-generating rather than providing definitive clinical guidance. Future trials should adopt standardized acupuncture protocols and uniform sham controls to validate the findings of this meta-analysis. Lastly, sham acupoints and sham acupuncture remain persistent dilemmas in acupuncture-related research. These two interventions may exert distinct effects. For instance, needling at non-acupoints or superficial needling may produce partial physiological responses, which may underestimate the true effect of acupuncture. Therefore, future studies should adopt uniform placebo or sham acupuncture controls to strengthen the current findings.

## Conclusions

Acupuncture may be associated with improvements in CPR, live birth rates, the number of optimal embryos, and E_2_ levels on the day of hCG, while reducing the total Gn dosage and duration of Gn treatment without elevating the risk of OHSS during IVF/ICSI. However, these findings are limited by high statistical heterogeneity, publication bias, lack of blinding, variable protocols, and geographic restriction. No causal conclusions can be drawn, and the observed associations do not confirm a direct effect of acupuncture on IVF/ICSI outcomes. Subgroup findings are exploratory and need verification. In conclusion, acupuncture may be a potential adjunctive option for women with PCOS undergoing IVF/ICSI, but definitive clinical recommendations are not supported. Further standardized multicenter RCTs are required to clarify these preliminary findings.

## Data Availability

The original contributions presented in the study are included in the article/**Supplementary Material**. Further inquiries can be directed to the corresponding authors.
