# Supplementary material for: Clinical efficacy of acupuncture for women with PCOS undergoing IVF/ICSI: a meta-analysis of randomized controlled trials
Source: Front Endocrinol (Lausanne). 2026 May 29;17:1845255. doi: 10.3389/fendo.2026.1845255 (PMC13259672; doi:10.3389/fendo.2026.1845255)
Supplement: Supplementary file 1 [file SupplementaryFile1.docx]

**The full search strategy**

**① PubMed = 19**

#1 "Fertilization in Vitro"[Mesh] OR "Sperm Injections, Intracytoplasmic"[Mesh] OR "Embryo Transfer"[Mesh] OR IVF OR in vitro fertilization OR intracytoplasmic sperm injection OR ICSI OR assisted reproductive technolog* OR ART OR "frozen embryo transfer" OR "frozen-thawed embryo transfer" OR "fresh embryo transfer" OR FET

#2 "Infertility, Female"[Mesh] OR infertile women OR female infertility OR subfertility OR subfertile

#3 "Polycystic Ovary Syndrome"[Mesh] OR PCOS OR polycystic ovary syndrome OR polycystic ovarian syndrome OR Stein-Leventhal syndrome

#4 Acupuncture OR Acupuncture Therapy[Mesh] OR acupunctur* OR electroacupuncture OR electro-acupuncture OR "manual acupuncture"

#5 "Randomized Controlled Trial"[Publication Type] OR "Randomized Controlled Trials as Topic"[Mesh] OR randomized controlled trial* OR randomised controlled trial* OR RCT OR random* OR trial OR "double blind" OR "double-blind"

#6 (#1 AND #2 AND #3)#7 (#4 AND #5 AND #6)

**② Cochrane Library = 24**

#1 "in vitro fertilization" OR IVF OR "intracytoplasmic sperm injection" OR ICSI OR (assisted NEXT reproductiv*) OR ART OR "frozen embryo transfer" OR "frozen-thawed embryo transfer" OR "fresh embryo transfer" OR FET OR "embryo transfer"

#2 "infertile women" OR "female infertility" OR subfertility OR subfertile OR sterility

#3 "polycystic ovary syndrome" OR PCOS OR "polycystic ovarian syndrome"

#4 acupuncture OR electroacupuncture OR "manual acupuncture"

#5 "randomized controlled trial" OR "randomised controlled trial" OR randomized OR randomised OR RCT OR "double blind" OR "double-blind" OR randomly OR "random allocation" OR "controlled clinical trial"

#6 (#1 AND #2) OR #1

#7 #3 AND #4 AND #5 AND #6

**③ Sinomed = 33**

#1 体外受精 OR IVF OR 卵胞浆内单精子注射 OR ICSI OR 辅助生殖 OR ART OR 冷冻胚胎移植 OR 冻融胚胎移植 OR 新鲜胚胎移植 OR FET OR 胚胎移植

#2 不孕女性 OR 女性不孕 OR 不育 OR 低生育力

#3 多囊卵巢综合征 OR PCOS

#4 针灸 OR 电针 OR 手针

#5 随机对照试验 OR 随机对照研究 OR 随机 OR RCT OR 双盲 OR 随机分配 OR 对照临床试验

#6 (#1 AND #2) OR #1

#7 #3 AND #4 AND #5 AND #6

**④ Web of Science = 35**

#1 "in vitro fertilization" OR IVF OR "intracytoplasmic sperm injection" OR ICSI OR "assisted reproductive technology" OR ART OR "frozen embryo transfer" OR "frozen-thawed embryo transfer" OR "fresh embryo transfer" OR FET OR "embryo transfer"

#2 "infertile women" OR "female infertility" OR subfertility OR subfertile OR sterility

#3 "polycystic ovary syndrome" OR PCOS OR "polycystic ovarian syndrome"

#4 acupuncture OR electroacupuncture OR "manual acupuncture"

#5 "randomized controlled trial" OR "randomised controlled trial" OR randomized OR randomised OR RCT OR "double blind" OR "double-blind" OR randomly OR "random allocation" OR "controlled clinical trial"

#6 (#1 AND #2) OR #1

#7 #3 AND #4 AND #5 AND #6

**⑤ Wanfang = 137**

主题:("不孕" OR "体外受精" OR "胚胎移植" OR "卵胞浆内单精子注射" OR ICSI OR "辅助生殖技术" OR IVF OR "冻融胚胎移植" OR "冷冻胚胎移植") and 主题:("多囊卵巢综合征" OR PCOS) and 主题:("针灸" OR "电针" OR "手针") and 主题:("随机对照试验" OR "随机" OR "RCT" OR "双盲")

**⑥ VIP = 196**

U=(("不孕" OR "体外受精" OR "胚胎移植" OR "卵胞浆内单精子注射" OR "ICSI" OR "IVF" OR "辅助生殖技术" OR "冻融胚胎移植") AND ("多囊卵巢综合征" OR "PCOS") AND ("针灸" OR "电针") AND ("随机对照试验" OR "随机" OR "RCT"))

**⑦ CNKI = 107**

TKA='体外受精'+'试管婴儿'+'卵胞浆内单精子注射'+'ICSI'+'辅助生殖技术'+'不孕'+'IVF'+'胚胎移植'+'冻融胚胎移植' and TKA='多囊卵巢综合征'+'PCOS' and TKA='针灸'+'电针'+'手针' and TKA='随机对照试验'+'随机'+'RCT'
