# Supplementary material for: Clinical efficacy of acupuncture for women with PCOS undergoing IVF/ICSI: a meta-analysis of randomized controlled trials
Source: Front Endocrinol (Lausanne). 2026 May 29;17:1845255. doi: 10.3389/fendo.2026.1845255 (PMC13259672; doi:10.3389/fendo.2026.1845255)
Supplement: Supplementary file 2 [file Table1.docx]

TABLE 1 Study main characteristics.

| **Study** | **Year** | **BMI (Kg/m^2^)** | | **Regimen of treatment** | | **No. of acupoints** | **Ovulation induction protocol** | **Duration of treatment** |
| --- | --- | --- | --- | --- | --- | --- | --- | --- |
|  |  | **Control group** | **Trial group** | **Control group** | **Trial group** |  |  |  |
| Chen (27) | 2009 | 23.71 ± 2.86 | 23.33 ± 2.61 | No | Electroacupuncture | 5 | GnRH agonist long protocol | The menstrual cycle prior to controlled ovarian hyperstimulation (COH) and the COH process itself |
| Li (28) | 2009 | 24.13 ± 3.34 | 24.66 ± 3.65 | No | Electroacupuncture | 5 | GnRH agonist long protocol | The menstrual cycle prior to COH and the COH process itself |
| Cui (29) | 2011 | 23.96 ± 3.14 | 24.24 ± 4.13 | No | Electroacupuncture | 5 | GnRH agonist long protocol | The menstrual cycle prior to COH and the COH process itself |
| Rashidi (30) | 2013 | 26.10 ± 4.15 | 27.83 ± 4.61 | No | Electroacupuncture | 14 | GnRH agonist long protocol | Start of downregulation, start of stimulation, 2 days before ovum pick-up, and immediately before and after embryo transfer |
| Yang (31) | 2015 | 23.33 ± 3.58 | 23.36 ± 6.40 | No | Electroacupuncture | 6 | GnRH agonist long protocol | The menstrual cycle prior to COH and the COH procedure |
| Li (32) | 2015 | 23 ± 4 | 24 ± 5 | No | Electroacupuncture | 6 | GnRH agonist long protocol | The menstrual cycle preceding COH and the COH phase |
| Liu (33) | 2019 | 23.23 ± 3.39 | 22.93 ± 3.01 | TCM | TCM + Manual acupuncture | 5 | Mild stimulation protocol | Three menstrual cycles |
| Cai (34) | 2020 | 22.95 ± 1.23 | 22.92 ± 1.22 | No | Manual acupuncture | 15 | GnRH antagonist protocol | Acupuncture was initiated on day 2 or 3 of menstruation, administered once every other day, three times per week, until the trigger day |
| Wu (35) | 2021 | 22.76 ± 3.65 | 22.56 ± 3.31 | No | Manual acupuncture | 8 | GnRH agonist long protocol | Acupuncture was initiated at the start of ovarian stimulation and administered for a total of three menstrual cycles |
| Xiang (36) | 2021 | 28.1 ± 6.3 | 27.3 ± 7.3 | Pseudo-acupuncture | Electroacupuncture | 10 | GnRH antagonist protocol | **The intervention was 25 min twice a week until the day of oocyte collection** |
| Guo (19) | 2022 | 24.12 ± 3.764 | 22.94 ± 3.169 | No | Electroacupuncture | 7 | GnRH agonist long protocol | Electroacupuncture intervention was added starting on the day of ovarian stimulation and continued until the day of oocyte retrieval |
| Xing (37) | 2022 | NM | NM | No | Manual acupuncture | 12 | NM | Three menstrual cycles |
| Wu (38) | 2022 | 22.66 ± 3.03 | 23.19 ± 2.59 | No | Manual acupuncture | 8 | GnRH agonist long protocol | All acupuncture procedures were applied during the menstrual cycle before controlled ovarian  hyperstimulation process.This treatment procedure was performed once every other day until the ovulation day for patients in the observation group |
| Liu (39) | 2022 | 28.93 ± 8.27 | 29.14 ± 7.81 | Pseudo-acupuncture | Electroacupuncture | 10 | GnRH agonist long protocol | Twice per week until the trigger day |
| Ren (40) | 2024 | 24.75 ± 1.27 | 24.90 ± 1.43 | No | Manual acupuncture | 14 | GnRH agonist long protocol | During the COH process, three times per week |
| Guan (41) | 2024 | 26.27 ± 1.85 | 26.68 ± 1.89 | Pseudo-acupuncture | Electroacupuncture | 9 | GnRH antagonist protocol | Electroacupuncture was initiated after the cessation of menstruation in the menstrual cycle prior to oocyte retrieval. It was administered twice per week until the trigger day |
| Xiao (17) | 2025 | 23.64 ± 3.55 | 23.59 ± 4.41 | Pseudo-acupuncture | Electroacupuncture | 5 | GnRH agonist protocol | Three months before embryo transfer |
| Pang (42) | 2025 | 27.75 ± 2.58 | 28.22 ± 2.75 | placebo acupuncture | Electroacupuncture | 8 | GnRH antagonist protocol | **The completion of menstruation in the previous oocyte retrieval cycle and continued until the day of oocyte retrieval (excluding the menstrual period), administered 2–3 times a week for a total of 6 sessions** |
| Yang (43) | 2025 | 23.82 ± 3.47 | 22.94 ± 1.74 | No | Manual acupuncture | 7 | GnRH antagonist protocol | Acupuncture was initiated after the cessation of menstruation one month prior to the IVF-ET cycle, administered once daily until the day before oocyte retrieval |
| Li (18) | 2025 | 24.10 ± 3.60 | 23.87 ± 3.68 | Pseudo-acupuncture | Electroacupuncture | 20 | GnRH agonist long protocol | Acupuncture was administered three times per week, with an interval of 2–4 days between sessions, for a total of 6–8 weeks (approximately 26 sessions). In addition, one acupuncture session was arranged within 1 hour before and one session within 1 hour after embryo transfer |
| Xin (44) | 2025 | 30.76 ± 0.85 | 30.40 ± 0.91 | Pseudo-acupuncture | Electroacupuncture | 5 | GnRH antagonist protocol | Initiated three menstrual cycles prior to oocyte retrieval |
| Liu (16) | 2026 | 22.350 (4.60) | 22.730 (4.49) | No | Manual acupuncture | 20 | GnRH antagonist protocol | **The first phase started on day 5 of the menstrual cycle before a month of COH, three times a week, every other day, 30 min each time, for a total of 12 times.The second phase started on day 3 of COH and received acupuncture treatment once a day for 30 min until the trigger day, for a total of 8 times** |

Abbreviations: TCM, Traditional Chinese medicine; NM, not mention.
